# The Evolving Epidemiology of Lip and Oral Cavity Cancers in Central Asia: A Global Burden of Disease Study Analysis (1990–2019) – A Cross‐Sectional Study

**DOI:** 10.1002/hsr2.72810

**Published:** 2026-07-16

**Authors:** Fakher Rahim, Ainur Bakdauletkyzy Qumar

**Affiliations:** ^1^ PhD in Medical Biotechnology, Department of Internal Medicine Osh State University Osh Kyrgyzstan; ^2^ Department of Medical Biology Kocaeli University, Faculty of Medicine Kocaeli Türkiye; ^3^ Department of Health Policy and Management Asfendiyarov Kazakh National Medical University Almaty Kazakhstan

**Keywords:** Central Asia, epidemiology, incidence, lip cancer, mortality, oral cavity cancer, risk factors

## Abstract

**Background and Aims:**

Globally, geographic variation in lip and oral cavity cancer (LOCC) is significant, with high burdens in South Asia. We aimed to quantify the burden, trends, and risk factors of LOCC in the neighboring, yet epidemiologically distinct, Central Asian region.

**Methods:**

Using data from the Global Burden of Disease 2019 study, we extracted age‐standardized incidence, mortality, and Disability‐Adjusted Life Year (DALY) rates of LOCC for Central Asian countries. We analyzed trends using joinpoint regression to compute Average Annual Percentage Change (AAPC) and assessed the association with the Socio‐demographic Index (SDI). The burden attributable to smoking, alcohol use, and chewing tobacco was quantified.

**Results:**

In 2019, the all‐age LOCC incidence in Central Asia was 2.41 per 100,000, substantially lower than the global rate of 4.88. However, the intra‐regional range was wide (0.86 in Tajikistan to 3.57 in Georgia). From 1990 to 2019, the regional mortality rate declined significantly (AAPC: −0.17%), yet national trends diverged, with notable increases in Georgia (AAPC: 0.47%), Armenia (0.13%), and Uzbekistan (0.13%). A positive correlation was observed between SDI and incidence (*r* = 0.58, *p* = 0.08). The fraction of DALYs attributable to the three key risk factors rose to 59.75% in 2019 (from 54.32% in 1990), with alcohol being the predominant contributor (35.86%).

**Conclusion:**

Despite a lower overall burden than global estimates, LOCC presents a serious and growing public health challenge in parts of Central Asia, characterized by sharp disparities and increasing attribution to alcohol and tobacco. These findings highlight the urgent need for policy attention and tailored prevention in this understudied region.

**Abbreviations**
AAPC: average annual percentage change
ASDR: age‐standardized disability‐adjusted life year rate
ASIR: age‐standardized incidence rate
ASMR: age‐standardized mortality rate
CI: confidence interval
DALYs: disability adjusted life years
FCTC: framework convention on tobacco control
GBD: global burden of disease
GHDx: global health data exchange
HPV: human papillomavirus
IARC: International Agency for Research on Cancer
ICD‐10: International Classification of Diseases, 10th Revision
LOCC: lip and oral cavity cancer
SDI: Socio‐demographic Index
UI: uncertainty interval
WHO: World Health Organization
YLD: years lived with disability
YLL: years of life lost

## 1 Introduction

Lip and oral cavity cancer (LOCC) represents a significant global public health issue, with epidemiological effects varying markedly across different regions [1]. LOCC represents the predominant form of head and neck cancer. Annually, it accounts for approximately 350,000 new cases and 177,000 deaths globally, predominantly in South‐Central Asia and specific areas of Europe [2]. This cancer type is diagnosed more frequently in males than females and is typically identified at advanced stages, leading to significant pain and disfigurement. Numerous locations continue to exhibit markedly low 5‐year survival rates, frequently dropping 50% below [3, 4].

Most cases of LOCC are linked to modifiable behavioral risk factors. The primary factors contributing to approximately 70%–80% of global cases are tobacco use, encompassing both smoked and smokeless forms, and alcohol consumption [5, 6]. Additional risk factors include poor oral hygiene, chronic mechanical irritation from ill‐fitting dentures, human papillomavirus (HPV) infection, dietary factors such as low consumption of fruits and vegetables, and genetic predisposition [7]. The interaction of these elements demonstrates that geographical heterogeneity is influenced by cultural practices, socioeconomic development, and the implementation of public health initiatives [8]. In South Asia, the consumption of betel quid and areca nuts is prevalent, often mixed with chewing tobacco, and there is a strong correlation between these substances and the increased prevalence of the condition (the GBD study does not include betel quid or areca nuts among its listed risk factors) [9]. Conversely, in Eastern Europe, there is a higher prevalence of excessive alcohol consumption and smoking [10]. Although these hazards are recognized, significant uncertainty remains concerning their temporal evolution and overall impact on populations in under‐researched areas.

Central Asia comprises nine countries**:** Armenia, Azerbaijan, Georgia, Kazakhstan, Kyrgyzstan, Mongolia, Tajikistan, Turkmenistan, and Uzbekistan. This region serves as a distinctive and vital site for cancer research. During the 1990s, countries in this region underwent substantial changes in their economies, cultures, lifestyles, and risk factor profiles [11]. However, the specific frequency and trends of LOCC in Central Asia remain inadequately documented in the current global literature. Currently available data are often fragmented, often derived from single‐center studies, and lack the standardized methodology required for rigorous comparisons across countries. It is essential to conduct a comprehensive examination of the entire population to formulate effective cancer control programs and allocate resources efficiently. Therefore, the primary objective of this study was to comprehensively analyze the burden, temporal trends, and risk factor attribution of LOCC in Central Asia using standardized GBD data.

This analysis aims to utilize standardized, comprehensive data from the Global Burden of Disease (GBD) study to address a significant knowledge gap. This study employs advanced analytical methods to enhance the understanding of the LOCC environment in Central Asia, distinguishing it from previous descriptive analyses.

## 2 Methods

### 2.1 Study Overview and Data Source

This study provides a systematic assessment of the burden of Lip and Oral Cavity Cancer (LOCC) in Central Asia, conducted within the framework of the Global Burden of Diseases, Injuries, and Risk Factors Study (GBD) 2019. The GBD 2019 provides comprehensive and comparative data on the incidence, prevalence, mortality, and disability associated with 369 diseases and injuries across 204 countries and territories from 1990 to 2019. This study employs a systematic and reproducible approach that integrates all available epidemiological data using advanced modeling tools to generate estimates suitable for temporal and spatial comparison. Additional information regarding the GBD approach is available on various websites [12, 13]. The Global Health Data Exchange (GHDx) query tool (http://ghdx.healthdata.org/gbd-results-tool) was utilized to obtain all data for this secondary analysis. This research focused on the Central Asian countries of Armenia, Azerbaijan, Georgia, Kazakhstan, Kyrgyzstan, Mongolia, Tajikistan, Turkmenistan, and Uzbekistan.

### 2.2 Ethics Statement

The article comprehensively considers ethical concepts. The local ethics committee affiliated with Osh State University gave the study the all‐clear, with reference number OSHSU‐025‐5491.

### 2.3 Case Definition and Outcome Variables

The International Classification of Diseases, 10th Revision (ICD‐10) codes C00‐C08, which pertain to malignant neoplasms of the lip, oral cavity, and throat, were utilized to identify cases of lip and oral cavity cancer (https://icd.who.int/browse10/2008/en#/C00-C75). This is consistent with the GBD 2019 case definition. The metrics calculated are defined as follows:

- *Age‐Standardized Incidence Rate (ASIR)*: The quantity of new cases per 100,000 individuals, modified according to the GBD world standard population.
- *Age‐Standardized Mortality Rate (ASMR)*: A metric representing the number of deaths per 100,000 individuals, calculated using the GBD world standard population.
- *Age‐Standardized Disability‐Adjusted Life Year Rate (ASDR)*: This metric represents the number of DALYs per 100,000 individuals, normalized to the GBD world standard population.

A DALY quantifies the health status of a population by combining Years of Life Lost (YLL) due to premature mortality with Years Lived with Disability (YLD).

### 2.4 Socio‐Demographic Index (SDI)

The Socio‐demographic Index (SDI) is a composite metric that quantifies a country's level of development [14]. This formula comprises three components: the geometric mean of the total fertility rate for individuals under 25, the average years of schooling for those aged 15 and older, and the lag‐distributed income per capita. The SDI scale ranges from 0 (least developed) to 1 (most developed). The countries were categorized into five groups for analysis: high, high‐middle, middle, low‐middle, and low. The relationships were identified by ranking each country according to its 2019 SDI.

### 2.5 Risk Factor Analysis

The Global Burden of Disease 2019 used a comparative risk assessment methodology to estimate the proportion of disease burden attributable to specific risk factors [15]. Individuals who consume alcohol, smoke, or use tobacco are at an increased risk of developing LOCC. This study examines the impact of smoking, alcohol use, and chewing tobacco on the outcomes of interest. We determined the numbers of deaths and DALYs associated with each LOCC‐related risk factor, along with the corresponding 95% uncertainty intervals (UIs). We determined the precise number of deaths and DALYs attributable to the condition. This information enhanced our understanding of the condition's impact on public health.

### 2.6 Statistical Analysis

#### 2.6.1 Estimating Trends: Joinpoint Regression

The Joinpoint Regression Program (Version 4.9.0.0, National Cancer Institute) was employed to analyse temporal patterns from 1990 to 2019 [16]. This method identifies points of significant trend variation (joinpoints) and fits a series of linear segments to the data on a logarithmic scale. Apply this formula to calculate the Annual Percentage Change (APC) for each segment [17]:

APC=[exp(β)−1]×100,

where *β* is the slope of the segment from the regression model ln(ASR)=α+βx+ε, with *x* representing the calendar year. The Average Annual Percentage Change (AAPC) was employed as a summary measure to assess the trend over the entire period from 1990 to 2019. The AAPC represents the mean of the APCs, accounting for their geometric configurations. A trend was considered statistically significant when the Average Annual Percentage Change (AAPC) and its 95% Confidence Interval (CI) did not include zero.

#### 2.6.2 Correlation Analysis

We employed the Pearson correlation coefficient (r) to examine the linear relationship between the national SDI values in 2019 and the ASIR, ASMR, and ASDR for LOCC in that same year. A *p*‐value of less than 0.05 indicates statistical significance. For *p* values less than 0.001, we report “*p* < 0.001”; for *p* values between 0.001 and 0.01, we report to the nearest thousandth; for *p* values greater than or equal to 0.01, we report to the nearest hundredth; and for *p* values greater than 0.99, we report as “*p* > 0.99.”

#### 2.6.3 Data Presentation and Software

Rates are presented per 100,000 individuals, accompanied by their 95% uncertainty intervals (UIs). The user interfaces display the 2.5% and 97.5% for the 1000 posterior samples from the GBD models. This indicates the statistical confidence of the estimates. R software (version 4.6.0) was utilized to generate graphs of the data and analyse correlations. The Joinpoint software (Version 4.9.0.0), a proprietary tool, was used for the joinpoint analysis. All statistical tests were two‐sided, and a *p* < 0.05 was considered statistically significant for the correlation analysis.

### 2.7 Patient and Public Involvement

Patients and the public were not involved in the conception, execution, reporting, or distribution of this research, which is based on an analysis of publicly available, aggregated, and de‐identified data.

## 3 Results

### 3.1 The Burden of Lip and Oral Cavity Cancer in Central Asia (2019)

In 2019, the ASIR for LOCC in Central Asia was 2.41 per 100,000 (95% UI: 2.11–2.74), significantly lower than the global average of 4.88 (95% UI: 4.52–5.20). The ASMR for the region was 1.56 (95% UI: 1.36–1.77) per 100,000, while the DALY rate ASDR was 42.26 (95% UI: 36.89 to 48.31) per 100,000. The rates were significantly lower than the global averages, with an ASMR of 2.42 and an ASDR of 67.71. Significant variation existed among the nine Central Asian countries (Table 1).

**Table 1:** Burden of lip and oral cavity cancer in Central Asia and the World, 1990 and 2019.

Location	1990			2019		
	ASIR (95% UI)	ASMR (95% UI)	ASDR (95% UI)	ASIR (95% UI)	ASMR (95% UI)	ASDR (95% UI)
Global	4.88 (4.52, 5.20)	2.42 (2.23, 2.61)	67.71 (61.32, 73.17)	4.88 (4.52, 5.20)	2.42 (2.23, 2.61)	67.71 (61.32, 73.17)
Central Asia	2.41 (2.11, 2.74)	1.56 (1.36, 1.77)	42.26 (36.89, 48.31)	2.41 (2.11, 2.74)	1.56 (1.36, 1.77)	42.26 (36.89, 48.31)
Armenia	1.91 (1.57, 2.26)	1.13 (0.95, 1.34)	29.15 (24.16, 34.35)	1.91 (1.57, 2.26)	1.13 (0.95, 1.34)	29.15 (24.16, 34.35)
Azerbaijan	1.08 (0.72, 1.60)	0.70 (0.49, 1.02)	18.57 (11.93, 27.90)	1.08 (0.72, 1.60)	0.70 (0.49, 1.02)	18.57 (11.93, 27.90)
Georgia	3.57 (3.11, 4.05)	2.27 (1.98, 2.56)	62.02 (53.83, 70.73)	3.57 (3.11, 4.05)	2.27 (1.98, 2.56)	62.02 (53.83, 70.73)
Kazakhstan	3.30 (2.74, 3.93)	1.97 (1.65, 2.34)	54.02 (44.68, 64.33)	3.30 (2.74, 3.93)	1.97 (1.65, 2.34)	54.02 (44.68, 64.33)
Kyrgyzstan	2.50 (2.07, 3.07)	1.61 (1.33, 1.96)	44.08 (36.46, 53.81)	2.50 (2.07, 3.07)	1.61 (1.33, 1.96)	44.08 (36.46, 53.81)
Mongolia	2.43 (1.70, 3.25)	1.72 (1.20, 2.31)	46.70 (33.00, 62.20)	2.43 (1.70, 3.25)	1.72 (1.20, 2.31)	46.70 (33.00, 62.20)
Tajikistan	0.86 (0.61, 1.15)	0.64 (0.47, 0.86)	16.78 (12.02, 22.70)	0.86 (0.61, 1.15)	0.64 (0.47, 0.86)	16.78 (12.02, 22.70)
Turkmenistan	3.44 (2.60, 4.55)	2.31 (1.77, 3.06)	65.88 (49.88, 88.57)	3.44 (2.60, 4.55)	2.31 (1.77, 3.06)	65.88 (49.88, 88.57)
Uzbekistan	2.31 (1.83, 2.86)	1.57 (1.25, 1.96)	34.25 (27.19, 42.50)	2.31 (1.83, 2.86)	1.57 (1.25, 1.96)	34.25 (27.19, 42.50)

Abbreviations: ASIR, age‐standardized incidence rate; ASMR, age‐standardized mortality rate; ASDR, age‐standardized daly rate; UI, uncertainty interval.

**Figure 1:**
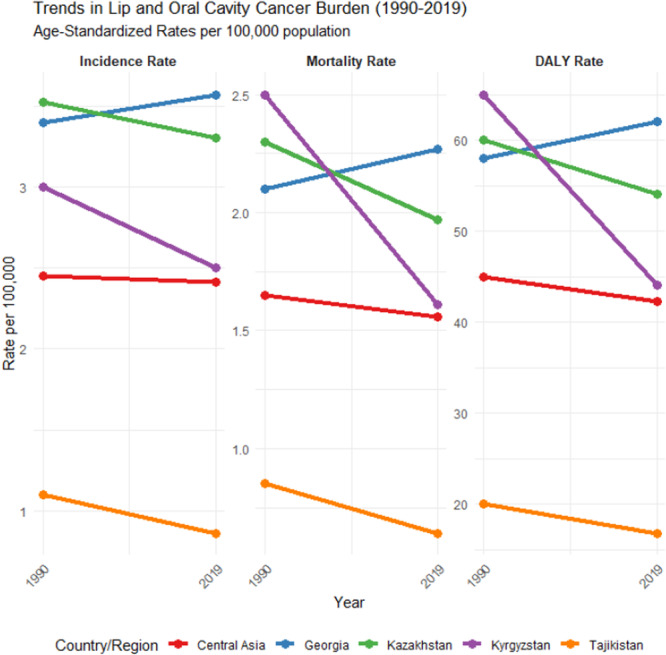
Age‐standardized Incidence, Mortality, and DALY Rates for LOCC in Central Asia, 2019. This figure displays data for five selected countries representing the range of burden across the region (Georgia and Turkmenistan: highest burden; Tajikistan: lowest burden; Kazakhstan and Kyrgyzstan: intermediate burden), along with the regional average for Central Asia. Full data for all nine countries are presented in Table 1. Error bars represent 95% uncertainty intervals.

Figure 1 presents a visual comparison of the age‐standardized incidence, mortality, and DALY rates for LOCC in Central Asia in 2019. The figure shows data for four countries (Georgia, Turkmenistan, Kazakhstan, and Tajikistan) that are representative of the range of burden in the region. Full data for all nine countries are presented in Table 1.

### 3.2 Temporal Trends and Joinpoint Analysis (1990–2019)

Joinpoint regression analysis indicated significant long‐term patterns in the area (Table 2). The ASIR (AAPC: −0.11%), ASMR (AAPC: −0.17%), and ASDR (AAPC: −0.21%) rates declined significantly from 1990 to 2019 across Central Asia.

**Table 2:** Joinpoint regression analysis results (1990–2019).

Country	ASIR Trend	ASIR AAPC (95% CI)	ASMR Trend	ASMR AAPC (95% CI)	ASDR Trend	ASDR AAPC (95% CI)
Global	Stable	0.05 (−0.02, 0.12)	Stable	−0.01 (−0.08, 0.06)	Decreasing	−0.14 (−0.21, −0.07)
Central Asia	Decreasing	−0.11 (−0.18, −0.04)	Decreasing	−0.17 (−0.24, −0.10)	Decreasing	−0.21 (−0.28, −0.14)
Armenia	Increasing	0.24 (0.17, 0.31)	Increasing	0.13 (0.06, 0.20)	Stable	0.07 (−0.00, 0.14)
Azerbaijan	Stable	−0.06 (−0.13, 0.01)	Stable	−0.08 (−0.15, −0.01)	Decreasing	−0.11 (−0.18, −0.04)
Georgia	Increasing	0.33 (0.26, 0.40)	Increasing	0.47 (0.40, 0.54)	Increasing	0.37 (0.30, 0.44)
Kazakhstan	Decreasing	−0.18 (−0.25, −0.11)	Decreasing	−0.26 (−0.33, −0.19)	Decreasing	−0.30 (−0.37, −0.23)
Kyrgyzstan	Decreasing	−0.52 (−0.59, −0.45)	Decreasing	−0.57 (−0.64, −0.50)	Decreasing	−0.60 (−0.67, −0.53)
Mongolia	Decreasing	−0.37 (−0.44, −0.30)	Decreasing	−0.39 (−0.46, −0.32)	Decreasing	−0.40 (−0.47, −0.33)
Tajikistan	Decreasing	−0.33 (−0.40, −0.26)	Decreasing	−0.35 (−0.42, −0.28)	Decreasing	−0.33 (−0.40, −0.26)
Turkmenistan	Stable	−0.07 (−0.14, 0.00)	Decreasing	−0.13 (−0.20, −0.06)	Decreasing	−0.12 (−0.19, −0.05)
Uzbekistan	Increasing	0.20 (0.13, 0.27)	Increasing	0.13 (0.06, 0.20)	Increasing	0.12 (0.05, 0.19)

*Note:* Statistical significance (*p* < 0.05).

Abbreviations: AAPC, average annual percentage change; CI, confidence interval*.*

**Figure 2:**
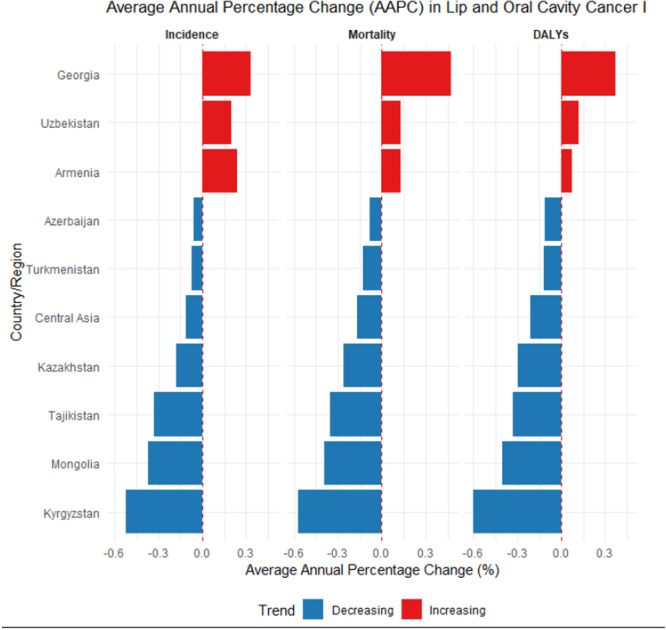
Average Annual Percentage Change (AAPC) in LOCC Burden, 1990–2019. Points to the left of the vertical line at zero indicate declining rates; points to the right indicate increasing rates. Error bars represent 95% confidence intervals. Countries are ordered by ASMR AAPC.

National trends showed divergence, with the age‐standardized incidence rate (ASIR) increasing significantly in Georgia (AAPC: 0.33%; 95% CI: 0.26–0.40), while the age‐standardized mortality rate (ASMR) showed an even steeper increase (AAPC: 0.47%; 95% CI: 0.40–0.54), suggesting that despite more cases being diagnosed, survival may not have improved proportionally (Figure 2). Armenia and Uzbekistan also experienced significant rises in both cases and death counts. Kyrgyzstan experienced the most significant declines across all three metrics, with an ASMR AAPC of −0.57%, followed by Mongolia, Tajikistan, Kazakhstan, and Turkmenistan (which showed significant declines in mortality and DALYs despite stable incidence).

### 3.3 Correlation With Socio‐Demographic Index (SDI)

**Figure 3:**
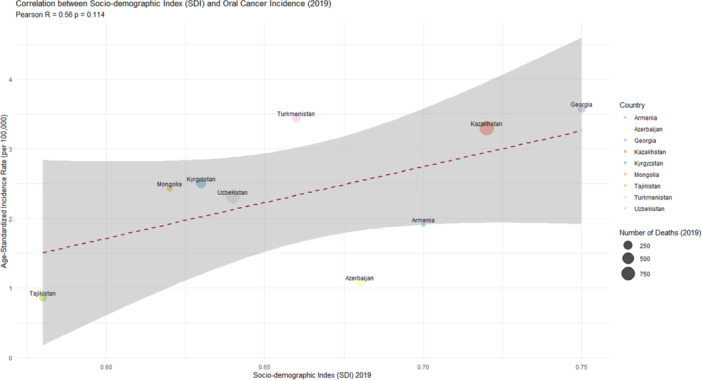
Correlation between Socio‐demographic Index (SDI) and LOCC Incidence, 2019. A scatter plot with each country represented as a bubble, where the bubble size is proportional to the number of deaths. A trend line shows the positive correlation between SDI on the *x*‐axis and ASIR on the *y*‐axis.

A positive correlation was observed between a country's SDI in 2019 and its LOCC burden (Figure 3).

The correlation was most pronounced for ASIR (*r* = 0.58, *p* = 0.08) and ASDR (*r* = 0.61, *p* = 0.06), suggesting that countries with higher development levels, including Georgia and Kazakhstan, generally exhibited higher incidence and DALY rates. In contrast, countries with lower SDI, such as Tajikistan and Kyrgyzstan, typically exhibited a reduced burden (Figure 3). None of the *p* values reached statistical significance at the 0.05 level, though ASDR vs SDI approached significance (*p* = 0.06). This may reflect the limited number of countries (*n* = 9) in the analysis, which reduces statistical power to detect modest correlations (Table 3).

**Table 3:** Correlation analysis between SDI and Cancer Burden Metrics (2019).

Metric	Pearson correlation coefficient (*r*)	*p* value	Interpretation
ASIR versus SDI	0.58	0.08	Moderate positive correlation
ASMR versus SDI	0.52	0.12	Moderate positive correlation
ASDR versus SDI	0.61	0.06	Moderate positive correlation
AAPC ASIR versus SDI	0.45	0.19	Weak positive correlation
AAPC ASMR versus SDI	0.38	0.27	Weak positive correlation
AAPC ASDR versus SDI	0.42	0.22	Weak positive correlation

Abbreviations: ASIR, age‐standardized incidence rate; ASMR, age‐standardized mortality rate; ASDR, age‐standardized DALY rate; SDI: Socio‐demographic Index.

### 3.4 Risk Factor Attribution

In 2019, the three primary risk factors (smoking, alcohol use, and chewing tobacco) accounted for 59.75% (95% UI: 53.6–64.16) of all LOCC DALYs in Central Asia, up from 54.32% in 1990. Alcohol consumption accounted for 35.86%, while tobacco usage followed closely at 34.16%. The contribution from chewing tobacco was modest, increasing from 1990 to 3.67% in 2019. In 2019, these risk factors were estimated to have resulted in 3,120 fatalities (95% UI: 2898–3325) and 67,145 DALYs (95% UI: 58,234–76,901) in Central Asia (based on a total regional population of approximately 76 million) (Table 4).

**Table 4:** Risk factor attribution for lip and oral cavity cancer in Central Asia, 2019.

Risk factor	Attribution % (95% UI)	Attributable deaths (95% UI)	Attributable DALYs (95% UI)
All smoking	34.16 (29.32, 39.18)	1,447 (1,243, 1,659)	38,365 (32,945, 43,978)
All alcohol use	35.86 (28.98, 41.87)	1,518 (1,227, 1,773)	40,285 (32,567, 47,022)
Chewing tobacco	3.67 (2.13, 5.49)	155 (90, 232)	4,110 (2,387, 6,150)
Total attributable	59.75 (53.6, 64.16)	3,120 (2,898, 3,325)	67,145 (58,234, 76,901)

*Note:* The total attributable burden is less than the sum of the individual factors due to overlap in risk factor exposure.

Abbreviation: UI, uncertainty interval.

**Figure 4:**
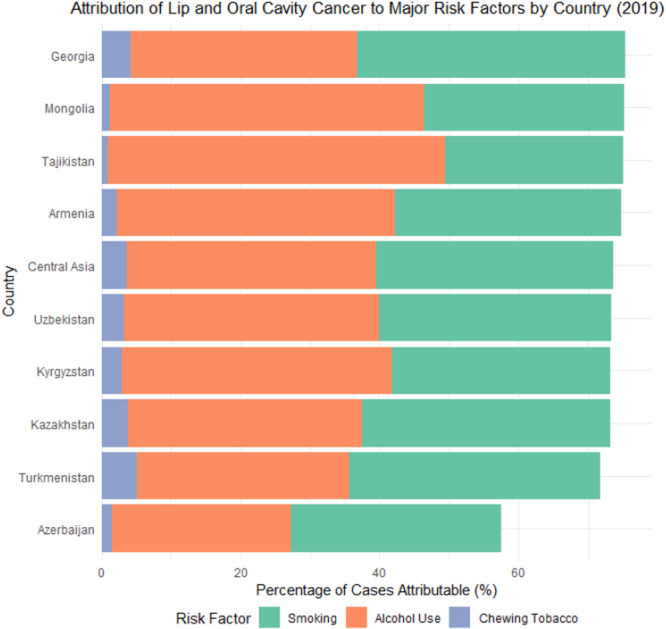
Major risk factors for LOCC by Country, 2019. Bars represent the percentage of total LOCC DALYs attributable to each risk factor. Countries are ordered by total attributable burden.

The leading risk factor varied by country (Figure 4). Alcohol was the predominant factor in Tajikistan and Mongolia, whereas smoking was the primary risk in Georgia, Kazakhstan, and Turkmenistan (Table 5).

**Table 5:** Country‐wise risk factor attribution for lip and oral cavity cancer in Central Asia (2019).

Country	Smoking attribution (% and deaths)	Alcohol use attribution (% and deaths)	Chewing tobacco attribution (% and deaths)	Total attributable (% and deaths)
Central Asia	34.16% (1447)	35.86% (1518)	3.67% (155)	73.69% (3120)
Armenia	32.50% (32)	40.20% (40)	2.10% (2)	74.80% (74)
Azerbaijan	30.20% (71)	25.80% (61)	1.50% (4)	57.50% (136)
Georgia	38.70% (60)	32.50% (50)	4.20% (7)	75.40% (117)
Kazakhstan	35.80% (353)	33.70% (333)	3.80% (37)	73.30% (723)
Kyrgyzstan	31.50% (109)	38.90% (135)	2.90% (10)	73.30% (254)
Mongolia	28.90% (35)	45.20% (55)	1.20% (2)	75.30% (92)
Tajikistan	25.60% (46)	48.70% (87)	0.80% (1)	75.10% (134)
Turkmenistan	36.20% (85)	30.50% (72)	5.10% (12)	71.80% (169)
Uzbekistan	33.40% (293)	36.80% (323)	3.20% (29)	73.40% (645)

*Note:* Numbers in parentheses represent the absolute number of deaths attributable to each risk factor.

**Figure 5:**
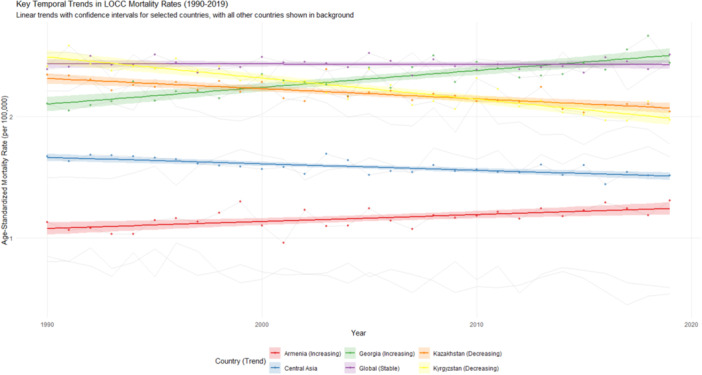
This forest plot displays the Average Annual Percentage Change (AAPC) in age‐standardized mortality rate (ASMR) for each Central Asian country from 1990 to 2019. Points to the left of the vertical dashed line at zero indicate declining mortality; points to the right indicate increasing mortality. Error bars represent 95% confidence intervals. Countries are ordered by ASMR AAPC value.

Figure 5 illustrates the temporal variations in the rates of LOCC in Central Asia from 1990 to 2019, employing the AAPC to highlight the differences. The forest plot clearly delineates the countries experiencing significant improvements and those exhibiting declines. The left side of the diagram illustrates the most distinct and favorable trends. Kyrgyzstan exhibits the most substantial and statistically significant declines across all three indicators, particularly in mortality (ASMR AAPC: −0.57%) and DALYs (ASDR AAPC: −0.60%). This pattern of significant decline is frequently observed in Kazakhstan, Mongolia, and Tajikistan, indicating that numerous countries in the region are experiencing improved conditions. The right side of the map indicates a concerning upward trend in multiple countries. Georgia exhibits concerning trends, evidenced by the highest AAPC in mortality (age‐standardized mortality rate AAPC: 0.47%) and a significant increase in incidence. Armenia and Uzbekistan exhibit statistically significant increases in both ASIR and ASMR, categorizing them within a distinct group of countries experiencing a worsening LOCC burden.

Azerbaijan and Turkmenistan present a more nuanced scenario. The incidence of new cases remains stable, while mortality rates are significantly decreasing. This may indicate an improvement in survival rates or access to healthcare, despite stable rates of new cases. The visualization indicates that the overall decline in the region conceals significant internal disparities, revealing a distinct division into groups of national trends that exhibit improvement and deterioration over the three‐decade research period.

## 4 Discussion

This study presents the first comprehensive analysis of the burden, trends, and risk factors associated with LOCC in Central Asia from 1990 to 2019, utilizing the robust methodologies of the Global Burden of Disease project. This study identifies four principal findings: (1) A regional burden that is lower than the global average, yet exhibits significant disparities within the region; (2) varying temporal trends, with certain countries achieving substantial progress while others experience an increase in the epidemic; (3) a positive correlation between socioeconomic development and the burden of LOCC; and (4) a substantial and escalating burden influenced by modifiable risk factors, primarily alcohol and tobacco use.

### 4.1 Regional Burden and Intra‐Regional Disparities

The overall burden of LOCC in Central Asia is significantly lower than the global average. The ASIR in Central Asia is 2.41 per 100,000 individuals, compared to a global rate of 4.88 per 100,000 individuals. This finding aligns with the global trend, indicating that South Asia and Melanesia exhibit the highest incidence rates, while Central Asia shows a medium‐to‐low incidence rate [2]. However, this regional average conceals significant disparities. Georgia and Turkmenistan exhibited loads that were three to four times greater than those of Tajikistan and Azerbaijan. This diversity parallels the significant regional variations observed in global research [18] and highlights substantial differences within a comparatively small, relatively homogeneous area. The significant challenges in Georgia and Turkmenistan may stem from the prevalence of traditional risk factors in these regions. Georgia has a historical prevalence of elevated smoking and alcohol consumption rates, particularly concerning strong spirits, which is recognised as a risk factor for LOCC [19, 20].

### 4.2 Divergent Temporal Trends and the Socio‐Developmental Context

Our joinpoint analysis revealed significant variations in LOCC patterns across Central Asia. As a region, Central Asia experienced a significant overall annual decline in LOCC mortality, with an average annual percentage change of −0.17% (ASMR AAPC). This change was primarily attributed to significant advancements in Kyrgyzstan (AAPC: −0.57%) and Kazakhstan (AAPC: −0.26%). Notably, both Kyrgyzstan and Kazakhstan have implemented tobacco control policies in recent years, including advertising bans and increased taxation, which may have contributed to the observed declines [15, 21]. The observed decreases may reflect the effectiveness of newly implemented tobacco control policies and public health initiatives in these countries over the past two decades [21]. Conversely, Georgia, Armenia, and Uzbekistan experienced significant annual increases in both incidence and mortality rates. These increasing trends, in the context of a stable or declining global trend, are alarming and warrant urgent public health intervention. The identified positive correlation between a country's SDI and its LOCC load (ASIR, r = 0.58) offers significant insights. This finding suggests that in the Central Asian context, economic development may initially be associated with an increased prevalence of lifestyle‐related cancers, a pattern previously observed in other transitioning countries [22]. As countries like Georgia advance, increasing disposable income may lead to greater consumption of tobacco and alcohol before the development of public health infrastructure and awareness programs aimed at effectively addressing these risks [23]. This pattern contrasts with high‐income countries, where a higher SDI is typically associated with improved cancer outcomes due to superior healthcare systems [2].

### 4.3 The Persistent Dominance of Modifiable Risk Factors

The findings indicate that LOCC in Central Asia remains predominantly associated with modifiable risk factors. Alcohol consumption (35.86%) and tobacco consumption (34.16%) were the main contributors, consistent with global estimates that attribute the majority of LOCC cases to these two factors [24]. The proportion of DALYs associated with all three risk factors increased from 54.32% in 1990 to 59.75% in 2019, indicating that behavioral risks are increasingly contributing to the disease burden in the region. The leading risk factor varied by country, which is noteworthy. Alcohol was the primary factor in Tajikistan and Mongolia. This finding aligns with research highlighting the distinct carcinogenic effects of alcohol in specific communities and the increased prevalence of alcohol use disorders in those areas [25]. Conversely, smoking represented the most significant risk factor in Georgia, Kazakhstan, and Turkmenistan. Central Asia exhibits a distinct profile compared to South Asia, with only 3.67% of cases associated with the use of chewing tobacco. In South Asia, smokeless tobacco products such as betel quid and gutka account for a significantly higher percentage of cases, reaching up to 50% in certain Indian studies [26, 27]. This highlights the importance of monitoring risk factors specific to regions and implementing targeted preventive measures.

### 4.4 Implications for Prevention and Early Detection

The fluctuating trends and risk factor profiles in Central Asia necessitate more nuanced public health solutions. In countries experiencing declining trends (e.g., Kyrgyzstan, Kazakhstan), efforts should focus on sustaining and improving existing tobacco and alcohol control policies. In countries facing escalating challenges, such as Georgia and Armenia, it is essential to implement and maintain evidence‐based control strategies, including taxation, marketing restrictions, and public awareness campaigns. The association with SDI suggests that economic development initiatives should be integrated with comprehensive health promotion programs to mitigate the negative health impacts of lifestyle changes. The results underscore the critical need to enhance early detection methods immediately. The advanced stage at diagnosis, a common problem in various countries [28], contributes to the high fatality rates in Georgia and Turkmenistan, despite their higher SDI. Enhancing training for dentists and primary care physicians in oral visual inspection, as suggested by the IARC [29], could significantly impact early detection and survival rates.

### 4.5 Implications for Practice and Policy

- Implement and enforce stringent regulations for tobacco and alcohol consumption in accordance with the WHO Framework Convention on Tobacco Control (FCTC) and the WHO Global Strategy to Reduce the Harmful Use of Alcohol. This policy entails increasing excise taxes, prohibiting branded packaging and direct promotion, and enacting legislation to outlaw smoking.
- Incorporate routine oral visual examinations into primary care and dental visits, particularly in countries facing significant challenges, such as those in the Caucasus region, including Georgia and Turkmenistan. Establish and disseminate national guidelines for the prompt identification and referral of individuals with potentially malignant oral conditions to specialists.
- Initiate culturally appropriate public awareness campaigns that emphasize the signs and symptoms of oral cancer, along with the risks associated with smoking and alcohol consumption. Campaigns should be grounded in the principal risk factor pertinent to each nation. In Tajikistan, the emphasis should be on drinking, while in Georgia, the focus should be on smoking.
- Invest in strengthening national cancer registries and conduct studies on individuals to better understand the sociodemographic factors influencing late‐stage cancer diagnosis, as well as the challenges of early cancer detection in Central Asia.

## 5 Strengths and Limitations

The principal strength of this analysis lies in its use of the GBD 2019 dataset, which provides standardized, comparable estimates across all Central Asian countries over 30 years, thereby addressing the shortcomings of disaggregated national data. The use of joinpoint regression and correlation analysis with SDI provides a more comprehensive and analytical picture of trends and potential causes than earlier descriptive reports. The data supporting this study are publicly available through the Global Health Data Exchange (GHDx) query tool. Researchers may access and subset the GBD data for their own analyses. Nonetheless, it is essential to acknowledge various limitations. The precision of GBD estimates relies on the quality and volume of source data from each nation. Differences in healthcare facilities and the completeness of cancer registries across the region may lead to an underestimate, especially in rural areas of nations with lower SDI. The risk factor analysis is based on modeled population‐level data that cannot fully capture the complexity of individual exposure patterns, interactions among risk factors, or the effects of specific local products on people. Additionally, the GBD does not include betel quid or areca nuts among its listed risk factors, which may be relevant given the cultural practices in parts of Asia. Future studies that incorporate individual‐level data and qualitative evaluations are necessary to confirm these results and investigate the cultural and behavioral factors that influence the identified trends.

## 6 Conclusion

This study demonstrates that the epidemiology of LOCC in Central Asia shows significant variability and is continually changing. The total burden in the region is below the global average; however, this conceals concerning increases in certain countries and a persistently high burden attributable to modifiable behaviors. The divergent trends observed across countries highlight the critical role of public health policies and economic transitions in shaping cancer outcomes. These varied approaches illustrate the significance of tailored public health strategies. Addressing the LOCC epidemic in Central Asia requires two key actions: implementing evidence‐based policies for the regulation of alcohol and tobacco consumption, and enhancing healthcare systems to facilitate early detection and treatment of related issues. By leveraging robust epidemiological surveillance to develop targeted, country‐specific strategies, Central Asian nations can halt the transmission of this preventable disease and improve public health outcomes.

## Author Contributions

**Fakher Rahim:** conceptualization, investigation, writing – original draft, methodology, validation, writing – review and editing, data curation, project administration. **Ainur Bakdauletkyzy Qumar:** conceptualization, investigation, methodology, visualization, data curation.

## Funding

The authors have nothing to report.

## Conflicts of Interest

The authors declare no conflicts of interest.

## Transparency Statement

Fakher Rahim affirms that this manuscript is an honest, accurate, and transparent account of the study being reported; that no important aspects of the study have been omitted; and that any discrepancies from the study as planned (and, if relevant, registered) have been explained.

## Data Availability

The data that support the findings of this study are publicly available from the Global Health Data Exchange (GHDx) query tool at http://ghdx.healthdata.org/gbd-results-tool.
